# An Exercise and Educational and Self-management Program Delivered With a Smartphone App (CareHand) in Adults With Rheumatoid Arthritis of the Hands: Randomized Controlled Trial

**DOI:** 10.2196/35462

**Published:** 2022-04-07

**Authors:** Pablo Rodríguez Sánchez-Laulhé, Luis Gabriel Luque-Romero, Francisco José Barrero-García, Ángela Biscarri-Carbonero, Jesús Blanquero, Alejandro Suero-Pineda, Alberto Marcos Heredia-Rizo

**Affiliations:** 1 Department of Physiotherapy Faculty of Nursing, Physiotherapy and Podiatry University of Seville Seville Spain; 2 Uncertainty, Mindfulness, Self, Spirituality (UMSS) Research Group University of Seville Seville Spain; 3 Research Unit, Distrito Sanitario Aljarafe-Sevilla Norte Andalusian Health Service Seville Spain; 4 Normal and Pathological Cytology and Histology Department University of Seville Seville Spain

**Keywords:** rheumatoid arthritis, telerehabilitation, self-management, mHealth, primary health care, physical therapy, exercise therapy, mobile applications, telehealth, health education, mobile phone

## Abstract

**Background:**

Rheumatoid arthritis (RA) is a prevalent autoimmune disease that usually involves problems of the hand or wrist. Current evidence recommends a multimodal therapy including exercise, self-management, and educational strategies. To date, the efficacy of this approach, as delivered using a smartphone app, has been scarcely investigated.

**Objective:**

This study aims to assess the short- and medium-term efficacy of a digital app (CareHand) that includes a tailored home exercise program, together with educational and self-management recommendations, compared with usual care, for people with RA of the hands.

**Methods:**

A single-blinded randomized controlled trial was conducted between March 2020 and February 2021, including 36 participants with RA of the hands (women: 22/36, 61%) from 2 community health care centers. Participants were allocated to use the CareHand app, consisting of tailored exercise programs, and self-management and monitoring tools or to a control group that received a written home exercise routine and recommendations, as per the usual protocol provided at primary care settings. Both interventions lasted for 3 months (4 times a week). The primary outcome was hand function, assessed using the Michigan Hand Outcome Questionnaire (MHQ). Secondary measures included pain and stiffness intensity (visual analog scale), grip strength (dynamometer), pinch strength (pinch gauge), and upper limb function (shortened version of the Disabilities of the Arm, Shoulder, and Hand questionnaire). All measures were collected at baseline and at a 3-month follow-up. Furthermore, the MHQ and self-reported stiffness were assessed 6 months after baseline, whereas pain intensity and scores on the shortened version of the Disabilities of the Arm, Shoulder, and Hand questionnaire were collected at the 1-, 3-, and 6-month follow-ups.

**Results:**

In total, 30 individuals, corresponding to 58 hands (CareHand group: 26/58, 45%; control group: 32/58, 55%), were included in the analysis; 53% (19/36) of the participants received disease-modifying antirheumatic drug treatment. The ANOVA demonstrated a significant time×group effect for the total score of the MHQ (*F*_1.62,85.67_=9.163; *P*<.001; *η^2^*=0.15) and for several of its subscales: overall hand function, work performance, pain, and satisfaction (all *P*<.05), with mean differences between groups for the total score of 16.86 points (95% CI 8.70-25.03) at 3 months and 17.21 points (95% CI 4.78-29.63) at 6 months. No time×group interaction was observed for the secondary measures (all *P*>.05).

**Conclusions:**

Adults with RA of the hands who used the CareHand app reported better results in the short and medium term for overall hand function, work performance, pain, and satisfaction, compared with usual care. The findings of this study suggest that the CareHand app is a promising tool for delivering exercise therapy and self-management recommendations to this population. Results must be interpreted with caution because of the lack of efficacy of the secondary outcomes.

**Trial Registration:**

ClinicalTrials.gov NCT04263974; https://clinicaltrials.gov/ct2/show/NCT04263974

**International Registered Report Identifier (IRRID):**

RR2-10.1186/s13063-020-04713-4

## Introduction

### Background

Rheumatoid arthritis (RA) is one of the most frequent systemic autoimmune diseases globally (approximately 1% of the population worldwide), with a higher prevalence in women [1]. In Spain, up to 430,000 adults aged >20 years have been estimated to have this disease [2]. RA results in tissue damage and chronic inflammation [3], especially in the small synovial joints of the hands and wrists [4]. Clinical presentation often involves musculoskeletal deficits; for example, hand deformities, pain, and reduced grip strength (GS) and pinch strength [5], which lead to functional and social limitations [6], along with a decline in work ability, productivity losses [7], and worse quality of life [8]. Together, these factors cause a substantial socioeconomic burden [9].

Pharmacological management of RA with disease-modifying antirheumatic drugs (DMARDs) and nonsteroidal anti-inflammatory drugs can help decrease RA-related symptoms and progression [8], although it may also induce serious adverse events [8] and largely increase health care costs [10]. Therefore, beneficial, safe, and cost-effective interventions must be implemented and prioritized in daily settings. As such, exercise therapy, supervised or at home, has been proposed as a suitable first-line approach for people with RA [10-12]. The current literature suggests, with inconclusive evidence [11], that exercise training programs for RA of the hands may improve the range of movement [13] and hand and upper limb functions [14,15]. In addition, they can reduce muscle weakness [16], pain intensity [17], and disease flare-ups [18] and eventually enhance the effects of DMARDs [15].

Long-term adherence to treatment is challenging and typically low in patients with RA [19]. This increases the risk of higher disability [20] and compromises the efficacy of the therapy [21]. Implementing strategies to solve this issue appears to be essential [11]. Several recommendations have been made, including an exercise diary to foster self-management and monitor treatment progression; for example, dose and intensity of exercises, establishing realistic therapy goals, a verbal or written commitment from the patient [12], providing information and education about the disease [12,19], and maintaining regular email or telephone contact [22], among others.

eHealth, as the use of communication technologies to support health-related fields, is a feasible solution for intervention delivery and has become imperative for health care systems, even more in the current COVID-19 pandemic context [23,24]. Mobile apps are the most effective eHealth modalities for reducing pain interference in chronic pain conditions [25]. Similarly, telehealth exercise programs have been shown to be an alternative to treat pain, physical function, and quality of life in people with physical disabilities [26,27]. Recent literature concludes that feedback-guided exercises delivered with a tablet increase function after carpal tunnel release [28] and hasten return to work in individuals with wrist, hand, or finger injuries [29]. In adults with RA, digital interventions using smartphone apps are promising to support clinical care and empower self-management [30], although they may not be effective unless designed including evidence-based strategies to promote adherence [31]. Preliminary findings demonstrate that digital apps that encourage self-management and allow for self-monitoring of the condition can improve health outcomes in patients with RA of the hands [32,33].

The CareHand app (Healthinn) was designed and developed with input from users and under the supervision of experts to meet the latest scientific evidence and the needs of patients and professionals, which is uncommon in existing mobile health apps [34]. This app emerges as a solution for telerehabilitation of patients with rheumatic hands and includes strategies to foster active self-management routines and long-term adherence, but it needs to be evaluated in the clinical setting.

### Objectives

This study aims to investigate the short- and medium-term effectiveness of a home therapeutic exercise program combined with general and self-management recommendations, as implemented with a mobile app (CareHand), compared with a usual care approach (exercise program and recommendations on a paper sheet) in people with RA of the hands. We hypothesized that hand function would improve more for participants who used the CareHand app.

## Methods

### Study Design

An experimental, longitudinal, parallel, controlled, and single-blinded randomized trial was conducted following the published protocol [35], prospectively registered at ClinicalTrials.gov (NCT04263974) and including an extended long-term follow-up of 6 months.

### Ethics Approval

The research protocol complied with the ethical guidelines of the Declaration of Helsinki and was approved by the Research Ethics Committee of the Virgen del Rocio and Virgen Macarena University Hospitals, Seville, Spain (code number PI_RH_2018).

### Participants

Participants aged ≥18 years and with a medical diagnosis of RA of the hands, wrists, or fingers, based on the American College of Rheumatology guidelines [36], were selected through data available from the digital medical records at the Health Districts Northern Seville and Aljarafe, Andalusian Health Service, Seville, Spain. Eligible individuals were contacted via telephone and asked to participate.

To be included in the study, participants had to have a disease history lasting for at least two years [36], report current pain and disability in the hands or wrists [37], and possess a smartphone with internet access. Exclusion criteria were previous hand fracture or surgery [37], waiting for upper limb surgery [15], steroid injection in the month before recruitment [19], pregnancy [38], and diagnosis of cognitive problems that may preclude the completion of the study protocol [39].

### Intervention Strategies

Participants in both groups were asked to perform their exercise intervention protocol at home 4 times a week for 3 months, with each training session programmed to last approximately 15 to 20 minutes. Several telephone follow-up calls were made during the trial to monitor adherence to the intervention and to solve eventual problems.

The control group underwent the conventional primary care approach of the public health system where the study was conducted. This consisted of providing a written exercise program and recommendations on a paper sheet, together with pictures and written explanations of upper limb strengthening and stretching exercises focusing on the hands, wrist, and finger joints.

Individuals in the CareHand group were asked to use the CareHand app. This digital solution has been developed under the guidance of health care professionals (physiotherapists and physicians) for its use on Android or iOS smartphones and comprises treatment and monitoring systems for the rehabilitation of people with rheumatic hands. The company responsible for the app has successfully implemented a tablet application (ReHand) for the treatment of traumatic injuries of the wrist, hand, and fingers [28,29], but no studies have investigated the efficacy of CareHand.

**Figure 1:**
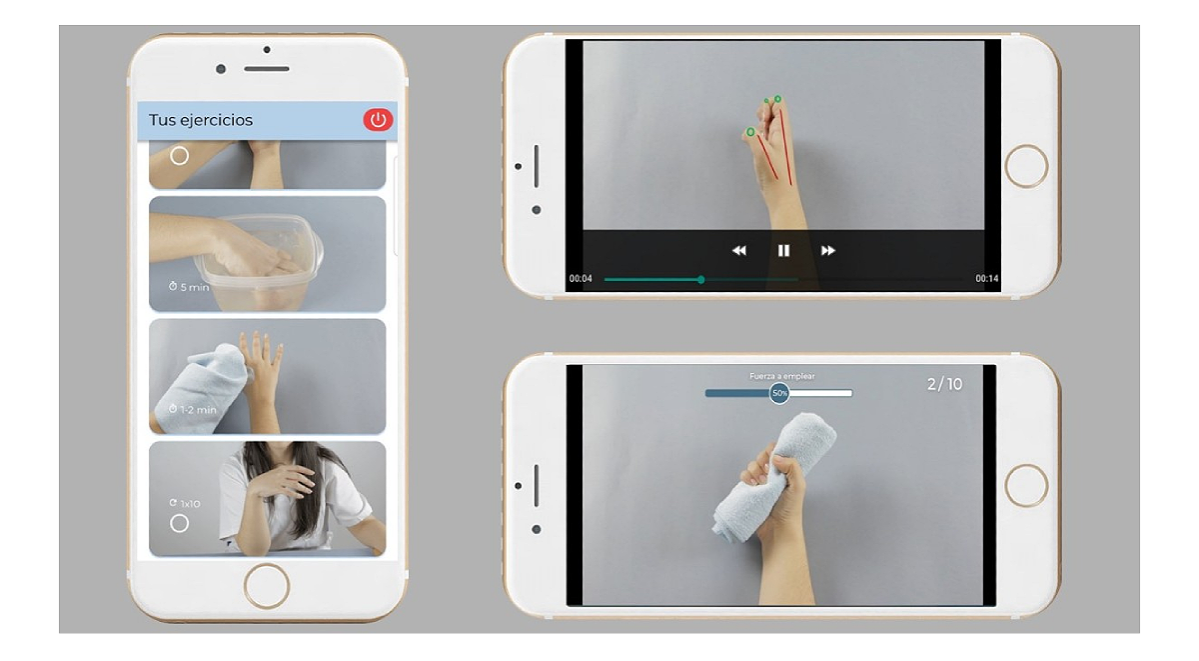
Exercise program of the CareHand app, including explanatory videos of mobility, strengthening, and stretching exercises.

**Figure 2:**
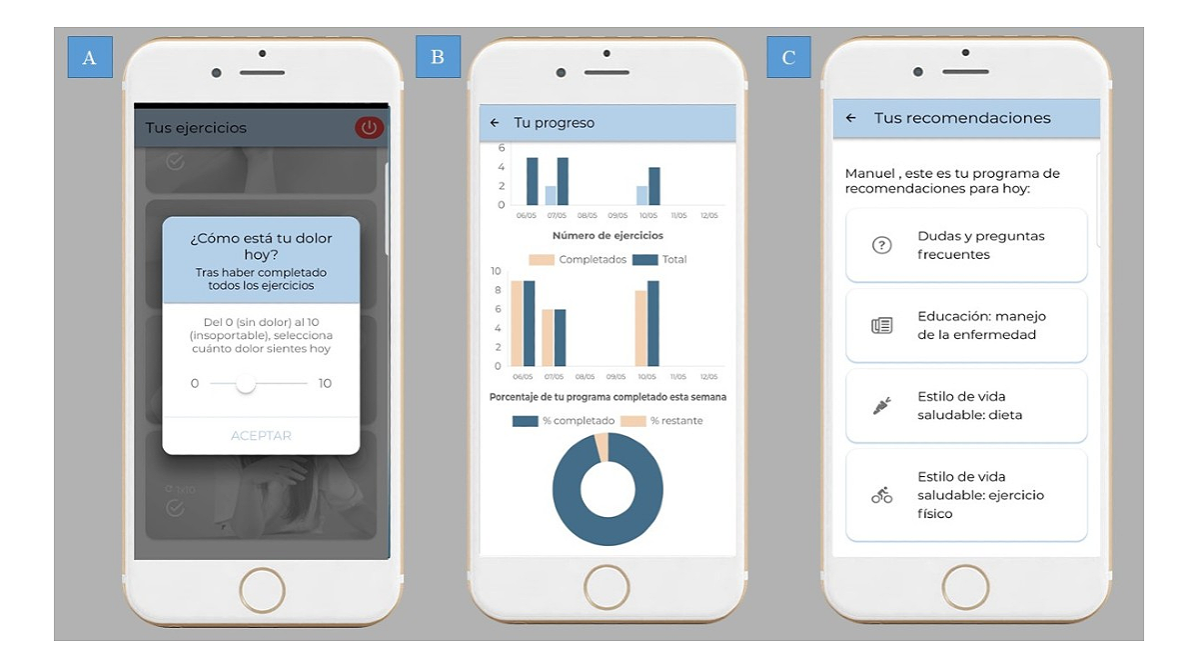
Features of the CareHand app. (A) Self-monitoring for pain intensity, (B) graphical representation of patient progress and adherence to exercises, and (C) educational advices section with information about joint protection and general recommendations.

The training program of the app, based on clinical guidelines for exercise therapy in people with RA of the hands [38,40], was delivered with explanatory videos, including a warm-up routine, and mobility, stretching, and strengthening exercises (Figure 1). Users of the app had to report their pain intensity twice a day (before and after the exercise program) and respond to self-reported outcome questionnaires once a week. The load and intensity of the exercises were automatically adapted to each participant’s pain intensity [41-43] (Figure 2). The CareHand app includes an exercise diary with a graphical representation of the progress in the planned treatment protocol and the evolution of pain intensity. This diary helps to promote positive self-management routines, monitor long-term adherence, and collect patient feedback [12,38]. The app also provides advice on diet and rules for joint protection. Educational and self-management strategies to handle RA-related symptoms and improve function during activities of daily living (ADL), along with recommendations for regular physical activity, were also provided (Figure 2) [19,38]. The app recorded and sent adherence charts to a cloud database. The data were monitored by the team member in charge of the interventions to ensure proper compliance with treatment.

### Enrollment

After contacted by telephone, those interested were scheduled for a face-to-face session at a community health center located at Camas or Sanlúcar la Mayor, Seville, Spain. During this session, a researcher (PRSL) assessed whether the individuals fulfilled the eligibility criteria. After agreeing to enroll, participants received further information about the trial and were asked to provide written informed consent. Then, clinical and demographic data were collected. After that, the participant was walked to a different room, and another assessor (LGLR, FJBG, or ABC), who was a general practitioner previously trained to evaluate the study measures, collected the outcomes at baseline: hand and upper limb function, self-reported pain intensity and stiffness, and GS and pinch strength. Following the baseline assessment, patients were randomly assigned to a study group using a random sequence in permuted blocks to allow a 1:1 distribution ratio. Sealed opaque envelopes were used to conceal intervention allocation. Participants were scheduled for a second appointment a week later, where the lead researcher (PRSL) explained the training protocol. This informative appointment was the starting session of the intervention.

### Outcome Measurements

#### Overview

Different measures were used to evaluate the efficacy of the interventions, including the Michigan Hand Outcome Questionnaire (MHQ) [44] for hand function; a visual analog scale (VAS) for self-reported pain and morning stiffness intensity [45,46]; a hydraulic hand dynamometer for GS; a pinch gauge for pinch strength [47]; and the shortened version of the Disabilities of the Arm, Shoulder, and Hand questionnaire (QuickDASH) [48] to measure upper limb function.

The lead investigator (PRSL) collected demographic and personal data at baseline, including age, gender, DMARDs consumption, and dominant hand.

#### Primary Outcome: Hand Function

The MHQ is an appropriate tool for individuals with chronic conditions of the hand. The questionnaire is divided into six subscales: function, ADL, work performance, pain, esthetic, and satisfaction [49]. Final scores ranged from 0 to 100, with higher values denoting better hand performance, except for pain [50]. The MHQ has shown high validity, reliability, and sensitivity in people with rheumatoid hands [44,50]. The Spanish version of the MHQ has good validity, reliability, and sensitivity to change [51]. This outcome was collected at baseline and at the 3- and 6-month follow-ups.

#### Secondary Outcomes

Participants reported their average pain and morning stiffness intensity in the previous week using a 11-point VAS. This measure shows good psychometric values when used to assess self-reported stiffness in patients with RA [45] and pain intensity in people with hand disorders [46]. Pain intensity was collected four times: at baseline and at the 1-, 3-, and 6-month follow-ups, whereas stiffness was collected at baseline and at the 3- and 6-month follow-ups.

A hand dynamometer (Saehan SH5001, Saehan Corp) was used, following the American Society of Hand Therapy statements, to evaluate GS [47]. Reporting GS is easy, quick, and reliable [52] and appears to be strongly related to the level of disability of rheumatic hands [53]. This procedure has demonstrated a great test-retest reliability; thus, measures were taken only once to avoid patient discomfort [54]. Maximum pain-free pinch force was assessed with a pinch gauge (13.5-kg mechanical pinch gauge, Baseline) [47], using a single measurement [55]. Pinch strength is inversely related to hand and upper limb function in patients with RA [56]. We collected GS and pinch strength data at baseline and at 3 months after the intervention.

Regarding upper limb function, the QuickDASH is a valid, reliable, easy to use, and widely used tool in patients with RA [5,48,57]. The questionnaire was completed at baseline and at the 1-, 3-, and 6-month follow-ups.

### Statistical Analysis

The sample size was calculated to achieve clinically significant differences (<13 points) [58], with a medium effect size (0.06≤*η^2^*≤0.14), for the overall hand function of the MHQ in the comparison between groups after intervention. RA is a symmetrical disease that involves joints bilaterally in over 60% of patients, although symptoms may [4] differ between sides [4]. Therefore, although the protocol estimation was made in terms of participants [35], we decided to deviate from the initial protocol and report the sample size in terms of the number of hands treated to adhere to common clinical practice. For an 80% desired power, an *α* value of .05, a correlation among repeated measures of 0.5, and a within-group variance of 10, a total of 56 hands were needed to complete the study (G*Power software, version 3.1.9.7; Kiel University).

Intention-to-treat principles were considered for all statistical analyses, which were conducted using SPSS Statistics (version 26; IBM Corp) software. Data are reported as mean (SD or 95% CI) or as percentages. The Shapiro-Wilk test was used to evaluate the normal distribution of the study measures. Mean outcome differences after intervention were compared using repeated-measures ANOVA, with group (control vs CareHand) as the between-subjects factor and time (baseline and the first-, third-, and sixth-month follow-ups) as the within-subjects factor. When the assumption of sphericity was violated, the Greenhouse-Heisser correction was applied. Partial eta squared values estimate the effect size. Statistical significance was set at *P*<.05.

## Results

### Flow of Participants

**Figure 3:**
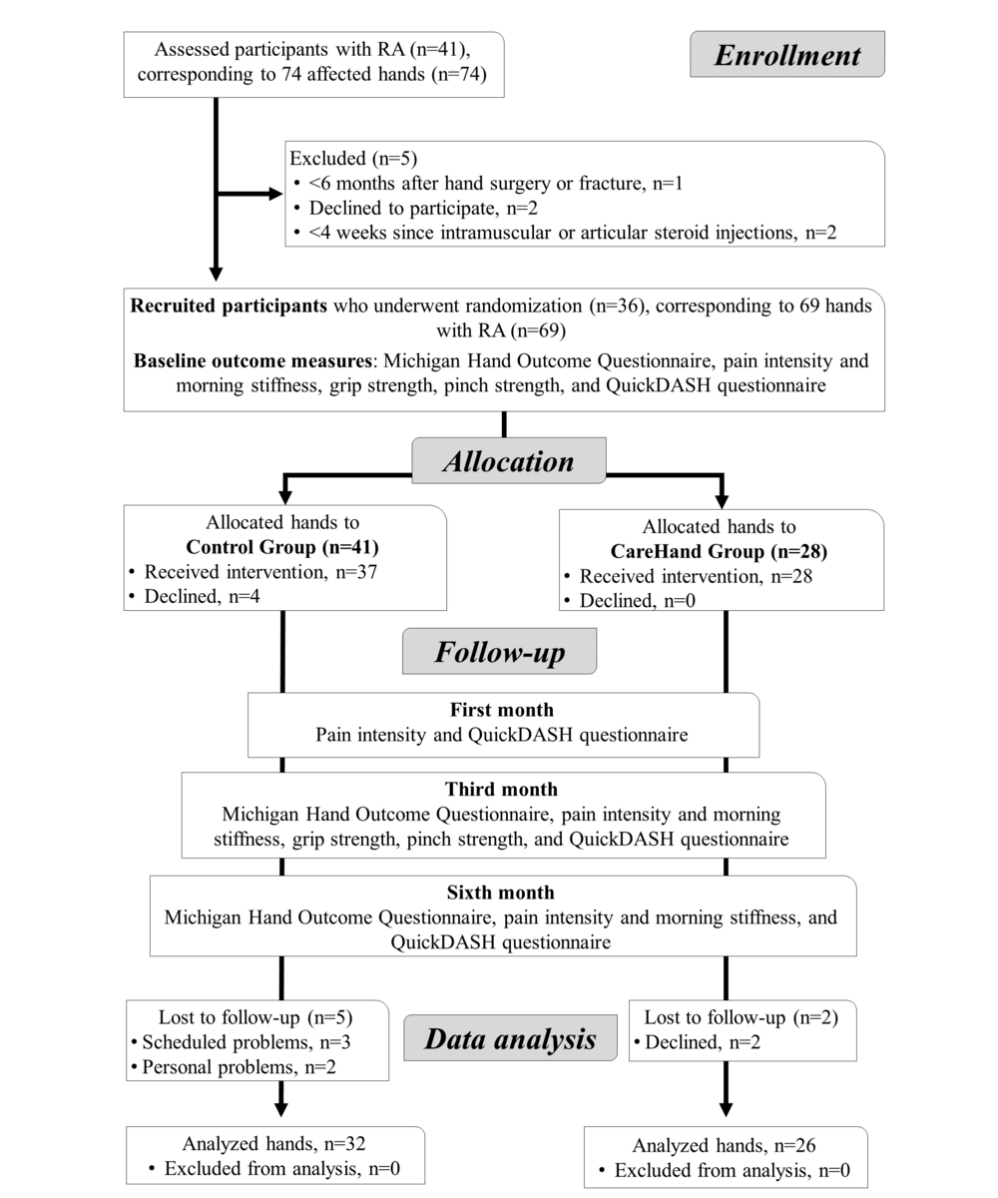
CONSORT (Consolidated Standards of Reporting Trials) flowchart of participants. QuickDASH: shortened version of the Disabilities of the Arm, Shoulder, and Hand questionnaire; RA: rheumatoid arthritis.

Between March 2020 and February 2021, a total of 41 adults with unilateral or bilateral RA of the hand were recruited. In those with a bilateral condition, both hands were selected if the inclusion and exclusion criteria were fulfilled. Of all 74 rheumatic hands, 5 (7%) were excluded at baseline, with 69 (93%) hands eligible for the study. Moreover, of the 36 recruited participants, 6 (17%) dropped out after baseline. Finally, 30 (83%) adults, corresponding to 58 hands (n=58), were included for statistical analysis. The flowchart of the participants is shown in Figure 3.

### Participant Characteristics

The baseline clinical and demographic characteristics of the participants are presented in Table 1. The patients were aged between 43 and 78 years, including 61% (22/36) women. A total of 92% (33/36) participants were right-handed, and 53% (19/36) participants were under pharmacological treatment with DMARDs. Moreover, 52% (36/69) of the hands that underwent intervention were right hands. Before the intervention, there were differences between the groups in the pain (*P*=.001) and satisfaction domains (*P*=.03) of the MHQ.

**Table 1:** Clinical and demographic baseline characteristics of participants.

			CareHand group (n=14, 28 hands)		Control group (n=22, 41 hands)		*P* value	
Age (years), mean (SD)			57.64 (7.25)		61.86 (10.76)		.21	
Gender (female), n (%)			9 (64)		13 (59)		.76	
DMARD^a^ treatment (yes), n (%)			10 (71)		9 (40)		.08	
**Dominant hand, n (%)**								.71
	Right	13 (93)		20 (91)				
	Left	0 (0)		2 (9)				
	Both	1 (7)		0 (0)				
Affected hand (right), n (%)			14 (50)		22 (54)		.77	
**MHQ,^b^ mean (SD)**								
	Overall hand function	50.89 (12.84)		57.81 (20.03)		.11		
	Activities of daily living	61.45 (22.94)		63.50 (31.66)		.77		
	Work performance	45 (29.25)		53.25 (35.47)		.32		
	Pain	62.50 (22.42)		39.02 (28.82)		.001		
	Esthetics	69.42 (28.28)		79.42 (29.32)		.16		
	Satisfaction	39.14 (21.41)		52.13 (25.38)		.03		
	Total score	50.57 (18.46)		61.09 (23.45)		.051		
Pain (VAS^c^; 0-10), mean (SD)			4.84 (2.76)		4.73 (2.79)		.87	
Stiffness (VAS; 0-10), mean (SD)			4.54 (2.86)		5.31 (3.41)		.33	
Grip strength (kg), mean (SD)			14.37 (7.91)		16.74 (9.65)		.29	
Pinch strength (kg), mean (SD)			4.15 (1.80)		4.52 (1.91)		.43	
QuickDASH^d^ (0-100), mean (SD)			52.27 (15.36)		43.05 (28.86)		.28	

^a^DMARD: disease-modifying antirheumatic drug.

^b^MHQ: Michigan Hand Outcome Questionnaire.

^c^VAS: visual analog scale.

^d^QuickDASH: shortened version of the Disabilities of the Arm, Shoulder, and Hand questionnaire.

### Hand Function

For the primary outcome, the ANOVA demonstrated a significant time×group effect for the total score of the MHQ (*F*_1.62,85.67_=9.163; *P*<.001; *η^2^*=0.15), with mean differences between groups of 16.86 points (95% CI 8.70-25.03) at the third-month follow-up and 17.21 points (95% CI 4.78-29.63) at the sixth-month follow-up. Furthermore, a statistically significant time×group effect was observed for several subscales of the MHQ: overall hand function (*F*_2,106_=3.298; *P*=.04; *η^2^*=0.06), work performance (*F*_2,98_=6.892; *P*=.002; *η^2^*=0.12), pain (*F*_2,106_=13.918; *P*<.001; *η^2^*=0.21), and satisfaction (*F*_1.69,89.77_=5.949; *P*=.006; *η^2^*=0.10; Table 2). The graphical representation of the mean differences in the MHQ in the 2 groups and in the different assessment points is included in Multimedia Appendix 1.

**Table 2:** Within- and between-group differences for the Michigan Hand Outcome Questionnaire (MHQ).

MHQ subscales			Within-groups differences from baseline, mean difference (95% CI)				Differences between CareHand and control groups					Time-group effect, *P* value	
			CareHand group		Control group		Values, mean difference (95% CI)		*P* value				
**Overall hand function**													.04
	Third month	11.67 (5.18 to 18.16)		−1.69 (−9.69 to 6.31)		13.36 (2.85 to 23.87)		.01					
	Sixth month	7.50 (−1.82 to 16.82)		0.31 (−7.72 to 8.35)		7.19 (−4.79 to 19.16)		.23					
**Activities of daily living**													.26
	Third month	11.55 (3.85 to 19.25)		5.59 (−2.70 to 13.87)		5.66 (−5.38 to 17.31)		.30					
	Sixth month	9.83 (2.12 to 17.55)		3.19 (−5.14 to 11.52)		6.64 (−4.68 to 17.97)		.25					
**Work performance**													.006
	Third month	10.00 (−2.94 to 22.94)		−0.97 (−10.94 to 9.00)		10.97 (−4.70 to 26.64)		.17					
	Sixth month	18.33 (2.40 to 34.26)		−5.50 (−16.12 to 5.12)		23.83 (5.77 to 41.90)		.01					
**Pain^a^**													<.001
	Third month	−22.50 (−33.5 to −11.49)		12.58 (1.59 to 23.57)		−35.08 (−50.54 to −19.62)		<.001					
	Sixth month	−17.31 (−27.32 to −7.30)		8.75 (−0.82 to 18.32)		−26.06 (−39.69 to −12.42)		<.001					
**Esthetic**													.10
	Third month	−0.78 (−12.77 to 11.21)		−11.28 (−22.80 to 0.23)		10.50 (5.93 to 26.93)		.21					
	Sixth month	2.40 (−16.88 to 21.68)		−18.16 (−32.38 to −3.93)		20.56 (−2.37 to 43.48)		.08					
**Satisfaction**													.006
	Third month	20.14 (10.33 to 29.96)		−3.23 (−10.93 to 4.47)		23.37 (11.37 to 35.36)		<.001					
	Sixth month	14.59 (0.30 to 28.88)		−4.04 (−14.02 to 5.94)		18.62 (2.04 to 35.20)		.02					
**Total**													<.001
	Third month	12.51 (5.48 to 19.55)		−4.35 (−9.32 to 0.62)		16.86 (8.70 to 25.03)		.001					
	Sixth month	11.56 (1.88 to 21.24)		−5.65 (−13.97 to 2.68)		17.21 (4.78 to 29.63)		.007					

^a^In the MHQ pain subscale, higher scores represent worse pain status.

### Secondary Outcomes

Scores for the secondary measures are presented in Table 3. The ANOVA reported no time×group interaction for any of the following outcomes: pain intensity (*F*_3,153_=1.352; *P*=.26; *η^2^*=0.03), morning stiffness (*F*_2,106_=1.299; *P*=.28; *η^2^*=0.02), GS (*F*_1,35_=0.001; *P*=.99; *η^2^*=0.001) and pinch strength (*F*_1,35_=0.112; *P*=.74; *η^2^*=0.003), and the QuickDASH (*F*_3,75_=0.924; *P*=.43; *η^2^*=0.04).

**Table 3:** Within- and between-group differences for self-reported pain and stiffness, grip strength and pinch strength, and upper limb function.

			Within-groups differences from baseline, mean difference (95% CI)					Differences between CareHand and control groups					Time-group effect, *P* value	
			CareHand group		Control group		Values, mean difference (95% CI)		*P* value					
**Pain intensity (VAS^a^)**														.26
	First month	0.94 (−0.36 to 2.25)		−0.20 (−1.45 to 1.06)		1.14 (−0.64 to 2.91)				.20				
	Third month	−0.44 (−1.42 to 0.54)		−0.32 (−1.39 to −0.75)		−0.12 (−1.58 to 1.34)				.87				
	Sixth month	0.90 (−0.43 to 2.24)		0.50 (−0.67 to 1.68)		0.40 (−1.33 to 2.13)				.65				
**Stiffness intensity (VAS)**														.28
	Third month	−0.38 (−1.89 to 1.14)		−0.95 (−1.81 to −0.09)		0.57 (−1.03 to 2.19)				.65				
	Sixth month	0.19 (−1.31 to 1.69)		−1.02 (−2.30 to 0.27)		1.21 (−0.71 to 3.13)				.21				
**Grip strength (kg)**														
	Third month	1.69 (−0.76 to 4.15)		1.69 (−0.35 to 3.73)		0.00 (−3.05 to 3.06)				.99	.99			
**Pinch strength (kg)**														
	Third month	−0.42 (−1.48 to 0.65)		−0.22 (−0.92 to 0.48)		−0.20 (−1.37 to 0.99)				.74	.74			
**QuickDASH^b^**														.43
	First month	−8.92 (−22.00 to 4.17)		−5.78 (−16.74 to 5.18)		−3.14 (−19.27 to 13.00)				.69				
	Third month	−10.80 (−22.47 to 0.87)		−7.05 (−15.75 to 1.63)		−3.74 (−17.25 to 9.77)				.57				
	Sixth month	−17.22 (−29.82 to −4.62)		−6.91 (−17.37 to 3.54)		−10.31 (−25.82 to 5.21)				.18				

^a^VAS: visual analog scale.

^b^QuickDASH: shortened version of the Disabilities of the Arm, Shoulder, and Hand questionnaire.

### Adverse Effects

No adverse events related to interventions were reported throughout the trial. A total of 17% (6/36) of participants experienced disease-associated pain flare-ups during follow-up.

## Discussion

### Principal Findings

As hypothesized, the findings of this study suggest that the CareHand app was better than conventional care in improving hand functional ability (overall hand function, work performance, pain, and satisfaction) in the short and medium term in adults with RA of the hands. However, no differences between groups were demonstrated for self-reported pain and morning stiffness, GS and pinch strength, and upper limb function; thus, the results must be interpreted cautiously.

### Hand Function

For the MHQ, the ANOVA showed a medium to large size effect in favor of the digital app. Mean differences between groups surpassed the clinically relevant thresholds for overall hand function (13 points) at the 3-month follow-up and for pain (11 points) at the 3- and 6-month follow-ups [58]. Pain and function are the best domains of the MHQ for identifying satisfied patients after treatment [58]. Recent evidence highlights the importance of considering hand function as the primary outcome in clinical trials investigating the efficacy of exercise therapy for RA of the hands [11], which should become a priority in daily practice [14].

The current literature on the impact of exercise on rheumatic hands is conflicting [11], although positive effects have mostly been reported [59]. Our findings are in line with those of a core trial on the topic, the Strengthening and Stretching for Rheumatoid Arthritis of the Hand (SARAH) project [15]. Lamb et al [15] concluded a positive effect on hand function (mean change: approximately 4.5 points), ADL, and total MHQ scores at 4 and 12 months after a multimodal approach with individualized exercises, strategies to enhance adherence, general recommendations, and joint protection education, as adjunct to drug treatment [15]. They also reported long-term improvements in hand dexterity. However, all observed changes only suggested a minimal clinical impact [15]. This study conducted an extended follow-up beyond 2 years, when the efficacy of the program diminished considerably compared with usual care [60]. Good results for hand function have also been reported when combining active hand exercises with wax baths [61]. In contrast, adding hand strengthening and mobility exercises to joint protection information was not superior to information alone in enhancing hand and finger function at 6 months [14]. Similarly, an 8-week exercise program, together with compensatory strategies; for example, joint protection, and use of assistive devices, added no benefits for task performance or ADL ability in women with RA of the hands [37]. These contradictory findings, along with the low quality of most trials on the topic, warrant new research [11].

eHealth has become an alternative and cost-saving approach to make evidence-based treatments available for patients and clinicians and to foster proactive self-management [30]. In this context, a web-based self-guided exercise program has been tested in adults with RA of the hands [62], with promising preliminary findings for hand function at 12 and 16 weeks [63]. Different mobile apps are also used by people with RA. However, most of them provide either symptom tracking or information alone and lack a comprehensive experience for patients [64]. Very recently, a mobile app including a structured hand exercise program was assessed for usability in adults with rheumatic hands, with good levels of satisfaction [65]. To date, the CareHand app is the first to be investigated in a clinical setting. This app includes educational information and adherence strategies, together with symptom tracking (exercise diary) through attractive audiovisual material [35]. The CareHand app also individualizes the exercise dose based on the self-reported pain intensity. This multimodal approach has proven to be effective in increasing hand function and self-efficacy in this population [15,19]. Higher self-efficacy is an empowerment protective factor in people with chronic symptoms and has been correlated with better hand function and quality of life in adults with RA [66]. All these app features may help to increase adherence to intervention [19] and treatment efficacy [11], even in the long run [21], which would explain our positive findings for the primary measure.

### Secondary Measures

According to the present evidence, systemic exercise treatment may be suitable for reducing RA-related pain [59]. However, it is still uncertain whether upper limb exercise therapy decreases hand pain or stiffness in adults with rheumatic hands [11]. In our study, the findings showed no effect of any of the interventions on self-reported pain or morning stiffness. Our results agree with those of previous trials where home exercise programs, alone or together with education and self-management routines, did not change pain intensity in the medium term [15,37,67,68] or long term [15,60] in this population. Similar findings were obtained when using digital technology to deliver an exercise regime [63]. In contrast, other trials have found that combining exercise with other forms of physical therapy helps to reduce hand pain, both in the short [16] and medium term [19,39,61,69]. RA is a condition that usually involves periods of flare-ups, which could affect self-reported pain scores and explain the conflicting evidence on the topic [70]. In addition, more than half of the participants (19/36, 53%) used DMARDs, which could also have influenced this outcome. Unsurprisingly, pain intensity did not improve despite changes in hand function. However, pain and function are not necessarily associated factors. This has been demonstrated in individuals with RA [37] and, most importantly, in people with chronic conditions [71] probably because of the multifactorial etiology of persistent pain [71]. Therefore, although therapists and patients with RA usually consider pain intensity as an important clinical measure [65], a recent systematic review proposed the use of function instead of pain as the primary outcome in this population [11]. Morning stiffness is one of the first symptoms caused by RA and is a predictor of a poor prognosis [45]. With regard to stiffness, evidence from the scientific literature is also contradictory, as observed for pain. Some studies have found good results for this outcome after exercise training [61], although most evidence points out the lack of efficacy of exercise programs for reducing morning hand stiffness [11,19,72]. The lack of treatment responsiveness has been partly explained by the great variability of this symptom within and between patients with RA [73].

Clinical and research guidelines recommend assessing GS and pinch strength in people with RA or osteoarthritis of the hands [74], as lower hand strength could be related to reduced functional ability [56] and greater structural joint damage [53]. The present literature is conflicting and unclear regarding this issue. Overall, very low–quality evidence indicates that hand exercise training, compared with no treatment, may improve GS and pinch strength in the short term [11]. However, when compared with usual care, it seems to have little or no benefit on hand strength in people with RA of the hands [11]. This could be a plausible explanation for our results. In line with this, joint protection programs [75] or general aerobic exercises [76] are not effective in increasing GS in patients with hand arthritis. Given the course of the disease and the heterogeneity of training protocols among studies, it is difficult to reach a definite conclusion. In addition, there are many different person-related factors; for example, age, gender, dominant hand, work occupation, leisure activities, and psychological aspects, that may influence this outcome in people with arthritic conditions, whether RA [53] or osteoarthritis [74]. Future studies should control for these confounding variables [53].

Finally, upper limb function is an important measure in RA, as it is associated with disease activity [19,77], self-efficacy [19], sensorimotor deficits [78], and quality of life [77]. However, there is little evidence regarding the effect of exercise training on this outcome. In the within-group analysis, we found improvements in both groups that surpassed the smallest detectable difference for the QuickDASH (6.9 points) [79] in the short and medium term. This may help to explain the lack of time×group effect, although differences between groups were clinically relevant at 6 months (−10.31 points, 95% CI −25.82 to 5.21 points). However, it has been questioned whether the QuickDASH is specific enough for people with RA of the hands [80]. Among the scarce studies in this area, Manning et al [19] delivered a similar intervention, using an Education, Self-Management, and Upper Extremity Exercise Training (EXTRA) program, with positive results for upper limb function at 12 weeks, compared with usual care, which eventually disappeared at 36 weeks, in line with former trials [68]. The scant evidence suggests a potential benefit of exercise therapy in decreasing upper limb disability, but further research is needed to support this statement.

### Limitations

This study has several limitations. The sample size was rather small, although relevant for the study aim and clinical purposes. In addition, there was a deviation from the initial protocol in terms of reporting the sample size estimation. This was intended to adhere pragmatically to the common standard practice of the clinical setting where the study was conducted. Despite the multicenter design, the participants were selected from a rural setting, which could limit the external validity of the findings. Changes in the study measures were evaluated in a medium-term follow-up (6 months); however, a long-term assessment is needed to better understand the efficacy of digital tools and the impact of the strategies used to engage patients. Important factors such as self-efficacy, psychological aspects, and treatment adherence were not measured. At baseline, both groups differed in the pain and satisfaction subscales of the MHQ, which may be a source of bias. In addition, owing to the restrictions imposed by the COVID-19 pandemic, some of the outcomes were collected via telephone. Finally, the normal course of RA includes periods of remission and exacerbation of symptoms, which can affect self-reported data.

### Clinical Implications

The CareHand app is a usable digital tool that opens a new field for the management of chronic rheumatic conditions of the hand. This app, developed with feedback from patients and health professionals, allows clinicians to treat people with RA and monitor their symptoms, evolution, and engagement with intervention. The app features foster proactive self-management. This may enhance self-efficacy and empower patients, which is key to managing chronic musculoskeletal disorders [81]. The feedback features also allow for a quick response if a disease flare-up appears [82]. The wide use of mobile devices and their portability represent a great potential impact of this app on health care delivery processes [65]. When implemented in a clinical setting, the CareHand app could reduce unnecessary visits to medical centers, as observed with other telehealth strategies in RA [83], with subsequent economic implications.

### Conclusions

A multimodal approach including a home exercise regime and self-management recommendations, as delivered with the CareHand smartphone app, was more effective than providing written instructions for the exercise program to improve hand function in the short and medium term in adults with RA of the hands. Despite these promising findings, no effects were found for self-reported pain intensity and stiffness, hand strength, and upper limb function.

## Appendix

Multimedia Appendix 1 Mean changes from baseline (A) to 3-month (B) and 6-month (C) follow-ups for the Michigan Hand Outcome Questionnaire (total and subscale scores).

Multimedia Appendix 2 CONSORT-EHEALTH checklist (V 1.6.1).

## Abbreviations

ADL: activities of daily living
DMARD: disease-modifying antirheumatic drug
EXTRA: Education, Self-Management, and Upper Extremity Exercise Training
GS: grip strength
MHQ: Michigan Hand Outcome Questionnaire
QuickDASH: shortened version of the Disabilities of the Arm, Shoulder, and Hand questionnaire
RA: rheumatoid arthritis
SARAH: Strengthening and Stretching for Rheumatoid Arthritis of the Hand
VAS: visual analog scale

## References

[1] Alamanos Y, Voulgari PV, Drosos AA (2006). Incidence and prevalence of rheumatoid arthritis, based on the 1987 American College of Rheumatology criteria: a systematic review. Semin Arthritis Rheum.

[2] Silva-Fernández L, Macía-Villa C, Seoane-Mato D, Cortés-Verdú R, Romero-Pérez A, Quevedo-Vila V, Fábregas-Canales D, Antón-Pagés F, Añez G, Brandy A, Martínez-Dubois C, Rubio-Muñoz P, Sánchez-Piedra C, Díaz-González F, Bustabad-Reyes S (2020). The prevalence of rheumatoid arthritis in Spain. Sci Rep.

[3] Zamanpoor M (2019). The genetic pathogenesis, diagnosis and therapeutic insight of rheumatoid arthritis. Clin Genet.

[4] Aletaha D, Smolen JS (2018). Diagnosis and management of rheumatoid arthritis: a review. JAMA.

[5] Palamar D, Er G, Terlemez R, Ustun I, Can G, Saridogan M (2017). Disease activity, handgrip strengths, and hand dexterity in patients with rheumatoid arthritis. Clin Rheumatol.

[6] Bergstra SA, Murgia A, Te Velde AF, Caljouw SR (2014). A systematic review into the effectiveness of hand exercise therapy in the treatment of rheumatoid arthritis. Clin Rheumatol.

[7] Chaudhari P (2008). The impact of rheumatoid arthritis and biologics on employers and payers. Biotechnol Healthc.

[8] Smolen JS, Aletaha D, McInnes IB (2016). Rheumatoid arthritis. Lancet.

[9] Verstappen SM (2015). Rheumatoid arthritis and work: the impact of rheumatoid arthritis on absenteeism and presenteeism. Best Pract Res Clin Rheumatol.

[10] Metsios GS, Kitas GD (2018). Physical activity, exercise and rheumatoid arthritis: effectiveness, mechanisms and implementation. Best Pract Res Clin Rheumatol.

[11] Williams MA, Srikesavan C, Heine PJ, Bruce J, Brosseau L, Hoxey-Thomas N, Lamb SE (2018). Exercise for rheumatoid arthritis of the hand. Cochrane Database Syst Rev.

[12] Hammond A, Prior Y (2016). The effectiveness of home hand exercise programmes in rheumatoid arthritis: a systematic review. Br Med Bull.

[13] Porter BJ, Brittain A (2012). Splinting and hand exercise for three common hand deformities in rheumatoid arthritis: a clinical perspective. Curr Opin Rheumatol.

[14] O'Brien AV, Jones P, Mullis R, Mulherin D, Dziedzic K (2006). Conservative hand therapy treatments in rheumatoid arthritis--a randomized controlled trial. Rheumatology (Oxford).

[15] Lamb SE, Williamson EM, Heine PJ, Adams J, Dosanjh S, Dritsaki M, Glover MJ, Lord J, McConkey C, Nichols V, Rahman A, Underwood M, Williams MA, Strengthening and Stretching for Rheumatoid Arthritis of the Hand Trial (SARAH) Trial Team (2015). Exercises to improve function of the rheumatoid hand (SARAH): a randomised controlled trial. Lancet.

[16] Buljina AI, Taljanovic MS, Avdic DM, Hunter TB (2001). Physical and exercise therapy for treatment of the rheumatoid hand. Arthritis Rheum.

[17] Katz P, Andonian BJ, Huffman KM (2020). Benefits and promotion of physical activity in rheumatoid arthritis. Curr Opin Rheumatol.

[18] Conigliaro P, Triggianese P, De Martino E, Fonti GL, Chimenti MS, Sunzini F, Viola A, Canofari C, Perricone R (2019). Challenges in the treatment of rheumatoid arthritis. Autoimmun Rev.

[19] Manning VL, Hurley MV, Scott DL, Coker B, Choy E, Bearne LM (2014). Education, self-management, and upper extremity exercise training in people with rheumatoid arthritis: a randomized controlled trial. Arthritis Care Res (Hoboken).

[20] O'Brien L (2012). The evidence on ways to improve patient's adherence in hand therapy. J Hand Ther.

[21] Cole T, Robinson L, Romero L, O'Brien L (2019). Effectiveness of interventions to improve therapy adherence in people with upper limb conditions: a systematic review. J Hand Ther.

[22] Roddy E, Zhang W, Doherty M, Arden NK, Barlow J, Birrell F, Carr A, Chakravarty K, Dickson J, Hay E, Hosie G, Hurley M, Jordan KM, McCarthy C, McMurdo M, Mockett S, O'Reilly S, Peat G, Pendleton A, Richards S (2005). Evidence-based recommendations for the role of exercise in the management of osteoarthritis of the hip or knee--the MOVE consensus. Rheumatology (Oxford).

[23] Eccleston C, Blyth FM, Dear BF, Fisher EA, Keefe FJ, Lynch ME, Palermo TM, Reid MC, de C Williams AC (2020). Managing patients with chronic pain during the COVID-19 outbreak: considerations for the rapid introduction of remotely supported (eHealth) pain management services. Pain.

[24] Berkovic D, Ackerman IN, Briggs AM, Ayton D (2020). Tweets by people with arthritis during the COVID-19 pandemic: content and sentiment analysis. J Med Internet Res.

[25] Slattery BW, Haugh S, O'Connor L, Francis K, Dwyer CP, O'Higgins S, Egan J, McGuire BE (2019). An evaluation of the effectiveness of the modalities used to deliver electronic health interventions for chronic pain: systematic review with network meta-analysis. J Med Internet Res.

[26] Dias JF, Oliveira VC, Borges PR, Dutra FC, Mancini MC, Kirkwood RN, Resende RA, Sampaio RF (2021). Effectiveness of exercises by telerehabilitation on pain, physical function and quality of life in people with physical disabilities: a systematic review of randomised controlled trials with GRADE recommendations. Br J Sports Med.

[27] Pastora-Bernal JM, Martín-Valero R, Barón-López FJ, Estebanez-Pérez MJ (2017). Evidence of benefit of telerehabitation after orthopedic surgery: a systematic review. J Med Internet Res.

[28] Blanquero J, Cortés-Vega MD, García-Frasquet MÁ, Sánchez-Laulhé PR, Nieto Díaz de Los Bernardos MI, Suero-Pineda A (2019). Exercises using a touchscreen tablet application improved functional ability more than an exercise program prescribed on paper in people after surgical carpal tunnel release: a randomised trial. J Physiother.

[29] Blanquero J, Cortés-Vega MD, Rodríguez-Sánchez-Laulhé P, Corrales-Serra BP, Gómez-Patricio E, Díaz-Matas N, Suero-Pineda A (2020). Feedback-guided exercises performed on a tablet touchscreen improve return to work, function, strength and healthcare usage more than an exercise program prescribed on paper for people with wrist, hand or finger injuries: a randomised trial. J Physiother.

[30] Dixon WG, Michaud K (2018). Using technology to support clinical care and research in rheumatoid arthritis. Curr Opin Rheumatol.

[31] Hamine S, Gerth-Guyette E, Faulx D, Green BB, Ginsburg AS (2015). Impact of mHealth chronic disease management on treatment adherence and patient outcomes: a systematic review. J Med Internet Res.

[32] Mollard E, Michaud K (2018). A mobile app with optical imaging for the self-management of hand rheumatoid arthritis: pilot study. JMIR Mhealth Uhealth.

[33] Seppen BF, Wiegel J, L'ami MJ, Duarte Dos Santos Rico S, Catarinella FS, Turkstra F, Boers M, Bos WH (2020). Feasibility of self-monitoring rheumatoid arthritis with a smartphone app: results of two mixed-methods pilot studies. JMIR Form Res.

[34] Voth EC, Oelke ND, Jung ME (2016). A theory-based exercise app to enhance exercise adherence: a pilot study. JMIR Mhealth Uhealth.

[35] Rodríguez-Sánchez-Laulhé P, Luque-Romero LG, Blanquero J, Suero-Pineda A, Biscarri-Carbonero Á, Barrero-García FJ, Heredia-Rizo AM (2020). A mobile app using therapeutic exercise and education for self-management in patients with hand rheumatoid arthritis: a randomized controlled trial protocol. Trials.

[36] American College of Rheumatology Subcommittee on Rheumatoid Arthritis Guidelines (2002). Guidelines for the management of rheumatoid arthritis: 2002 update. Arthritis Rheum.

[37] Ellegaard K, von Bülow C, Røpke A, Bartholdy C, Hansen IS, Rifbjerg-Madsen S, Henriksen M, Wæhrens EE (2019). Hand exercise for women with rheumatoid arthritis and decreased hand function: an exploratory randomized controlled trial. Arthritis Res Ther.

[38] Williams MA, Williamson EM, Heine PJ, Nichols V, Glover MJ, Dritsaki M, Adams J, Dosanjh S, Underwood M, Rahman A, McConkey C, Lord J, Lamb SE (2015). Strengthening and stretching for rheumatoid arthritis of the hand (SARAH). A randomised controlled trial and economic evaluation. Health Technol Assess.

[39] Rønningen A, Kjeken I (2008). Effect of an intensive hand exercise programme in patients with rheumatoid arthritis. Scand J Occup Ther.

[40] Williams MA, Heine PJ, Bruce J, Brosseau L, Lamb S (2012). Exercise therapy for the rheumatoid hand. Cochrane Database Syst Rev.

[41] Ageberg E, Link A, Roos EM (2010). Feasibility of neuromuscular training in patients with severe hip or knee OA: the individualized goal-based NEMEX-TJR training program. BMC Musculoskelet Disord.

[42] Sandal LF, Roos EM, Bøgesvang SJ, Thorlund JB (2016). Pain trajectory and exercise-induced pain flares during 8 weeks of neuromuscular exercise in individuals with knee and hip pain. Osteoarthritis Cartilage.

[43] Thomeé R (1997). A comprehensive treatment approach for patellofemoral pain syndrome in young women. Phys Ther.

[44] Chung KC, Pillsbury MS, Walters MR, Hayward RA (1998). Reliability and validity testing of the Michigan hand outcomes questionnaire. J Hand Surg Am.

[45] Barrios BI, Papasidero SB, Medina MA, Chaparro del Moral RE, Rillo OL, Paira SO, Sandobal C (2017). Correlación de diferentes métodos de valoración de la rigidez matinal con índices de actividad y discapacidad en pacientes con artritis reumatoidea. Rev Argent Reumatol.

[46] Castarlenas E, de la Vega R, Jensen MP, Miró J (2016). Self-report measures of hand pain intensity: current evidence and recommendations. Hand Clin.

[47] (1992). ASHT's clinical assessment recommendations. 2nd edition.

[48] Salaffi F, Di Carlo M, Carotti M, Farah S (2019). Validity and interpretability of the QuickDASH in the assessment of hand disability in rheumatoid arthritis. Rheumatol Int.

[49] Dritsaki M, Petrou S, Williams M, Lamb SE (2017). An empirical evaluation of the SF-12, SF-6D, EQ-5D and Michigan hand outcome questionnaire in patients with rheumatoid arthritis of the hand. Health Qual Life Outcomes.

[50] Waljee JF, Chung KC, Kim HM, Burns PB, Burke FD, Wilgis EF, Fox DA (2010). Validity and responsiveness of the Michigan hand questionnaire in patients with rheumatoid arthritis: a multicenter, international study. Arthritis Care Res (Hoboken).

[51] Miranda D, Ramírez J, Rueda L, García J, Wolf G, Lugo A LH (2008). Validación del "Michigan hand outcomes questionnaire" para población colombiana. Rev Colomb Reumatol.

[52] Sheehy C, Gaffney K, Mukhtyar C (2013). Standardized grip strength as an outcome measure in early rheumatoid arthritis. Scand J Rheumatol.

[53] Higgins SC, Adams J, Hughes R (2018). Measuring hand grip strength in rheumatoid arthritis. Rheumatol Int.

[54] Kennedy D, Jerosch-Herold C, Hickson M (2010). The reliability of one vs. three trials of pain-free grip strength in subjects with rheumatoid arthritis. J Hand Ther.

[55] MacDermid JC, Kramer JF, Woodbury MG, McFarlane RM, Roth JH (1994). Interrater reliability of pinch and grip strength measurements in patients with cumulative trauma disorders. J Hand Ther.

[56] Sferra da Silva G, de Almeida Lourenço M, de Assis MR (2018). Hand strength in patients with RA correlates strongly with function but not with activity of disease. Adv Rheumatol.

[57] Hammond A, Prior Y, Tyson S (2018). Linguistic validation, validity and reliability of the British English versions of the Disabilities of the Arm, Shoulder and Hand (DASH) questionnaire and QuickDASH in people with rheumatoid arthritis. BMC Musculoskelet Disord.

[58] Shauver MJ, Chung KC (2009). The minimal clinically important difference of the Michigan hand outcomes questionnaire. J Hand Surg Am.

[59] Sobue Y, Kojima T, Ito H, Nishida K, Matsushita I, Kaneko Y, Kishimoto M, Kohno M, Sugihara T, Seto Y, Tanaka E, Nakayama Y, Hirata S, Murashima A, Morinobu A, Mori M, Kojima M, Kawahito Y, Harigai M (2022). Does exercise therapy improve patient-reported outcomes in rheumatoid arthritis? A systematic review and meta-analysis for the update of the 2020 JCR guidelines for the management of rheumatoid arthritis. Mod Rheumatol.

[60] Williamson E, McConkey C, Heine P, Dosanjh S, Williams M, Lamb SE (2017). Hand exercises for patients with rheumatoid arthritis: an extended follow-up of the SARAH randomised controlled trial. BMJ Open.

[61] Dellhag B, Wollersjö I, Bjelle A (1992). Effect of active hand exercise and wax bath treatment in rheumatoid arthritis patients. Arthritis Care Res.

[62] Srikesavan C, Williamson E, Cranston T, Hunter J, Adams J, Lamb SE (2018). An online hand exercise intervention for adults with rheumatoid arthritis (mySARAH): design, development, and usability testing. J Med Internet Res.

[63] Srikesavan C, Williamson E, Thompson JY, Cranston T, Swales C, Lamb SE The online version of an evidence-based hand exercise program for people with rheumatoid arthritis: a mixed-method, proof-of-concept study. J Hand Ther..

[64] Luo D, Wang P, Lu F, Elias J, Sparks JA, Lee YC (2019). Mobile apps for individuals with rheumatoid arthritis: a systematic review. J Clin Rheumatol.

[65] Tonga E, Williamson E, Srikesavan C, Özen T, Sarıtaş F, Lamb SE (2021). A hand exercise mobile app for people with rheumatoid arthritis in Turkey: design, development and usability study. Rheumatol Int.

[66] Martinez-Calderon J, Meeus M, Struyf F, Luque-Suarez A (2020). The role of self-efficacy in pain intensity, function, psychological factors, health behaviors, and quality of life in people with rheumatoid arthritis: a systematic review. Physiother Theory Pract.

[67] Rubio Oyarzun D, Neuber Slater K, Araya Quintanilla F, Gutierrez Espinoza H, Arias Poblete L, Olguin Huerta C, Cornejo Cea M, Zagal Poblete J, Banda Maturana E (2017). Intervención de ejercicios de habilidad motora manual en el dolor y función en sujetos adultos con artritis reumatoide: serie de casos. Rev Soc Esp Dolor.

[68] Brorsson S, Hilliges M, Sollerman C, Nilsdotter A (2009). A six-week hand exercise programme improves strength and hand function in patients with rheumatoid arthritis. J Rehabil Med.

[69] Dogu B, Sirzai H, Yilmaz F, Polat B, Kuran B (2013). Effects of isotonic and isometric hand exercises on pain, hand functions, dexterity and quality of life in women with rheumatoid arthritis. Rheumatol Int.

[70] Wessel J (2004). The effectiveness of hand exercises for persons with rheumatoid arthritis: a systematic review. J Hand Ther.

[71] Martinez-Calderon J, Zamora-Campos C, Navarro-Ledesma S, Luque-Suarez A (2018). The role of self-efficacy on the prognosis of chronic musculoskeletal pain: a systematic review. J Pain.

[72] Brighton SW, Lubbe JE, van der Merwe CA (1993). The effect of a long-term exercise programme on the rheumatoid hand. Br J Rheumatol.

[73] Vliet Vlieland TP, Zwinderman AH, Breedveld FC, Hazes JM (1997). Measurement of morning stiffness in rheumatoid arthritis clinical trials. J Clin Epidemiol.

[74] Haugen IK, Aaserud J, Kvien TK (2021). Get a grip on factors related to grip strength in persons with hand osteoarthritis: results from an observational cohort study. Arthritis Care Res (Hoboken).

[75] Bobos P, Nazari G, Szekeres M, Lalone EA, Ferreira L, MacDermid JC (2019). The effectiveness of joint-protection programs on pain, hand function, and grip strength levels in patients with hand arthritis: a systematic review and meta-analysis. J Hand Ther.

[76] Hu H, Xu A, Gao C, Wang Z, Wu X (2021). The effect of physical exercise on rheumatoid arthritis: an overview of systematic reviews and meta-analysis. J Adv Nurs.

[77] Aktekin LA, Eser F, Başkan BM, Sivas F, Malhan S, Öksüz E, Bodur H (2011). Disability of arm shoulder and hand questionnaire in rheumatoid arthritis patients: relationship with disease activity, HAQ, SF-36. Rheumatol Int.

[78] Bearne LM, Coomer AF, Hurley MV (2007). Upper limb sensorimotor function and functional performance in patients with rheumatoid arthritis. Disabil Rehabil.

[79] Prodinger B, Hammond A, Tennant A, Prior Y, Tyson S (2019). Revisiting the disabilities of the arm, shoulder and hand (DASH) and QuickDASH in rheumatoid arthritis. BMC Musculoskelet Disord.

[80] Pedersini P, Negrini S, Cantero-Tellez R, Bishop MD, Villafañe JH (2020). Pressure algometry and palpation of the upper limb peripheral nervous system in subjects with hand osteoarthritis are repeatable and suggest central changes. J Hand Ther.

[81] Rice D, McNair P, Huysmans E, Letzen J, Finan P (2019). Best evidence rehabilitation for chronic pain part 5: osteoarthritis. J Clin Med.

[82] van Riel P, Alten R, Combe B, Abdulganieva D, Bousquet P, Courtenay M, Curiale C, Gómez-Centeno A, Haugeberg G, Leeb B, Puolakka K, Ravelli A, Rintelen B, Sarzi-Puttini P (2016). Improving inflammatory arthritis management through tighter monitoring of patients and the use of innovative electronic tools. RMD Open.

[83] de Thurah A, Stengaard-Pedersen K, Axelsen M, Fredberg U, Schougaard LM, Hjollund NH, Pfeiffer-Jensen M, Laurberg TB, Tarp U, Lomborg K, Maribo T (2018). Tele-health followup strategy for tight control of disease activity in rheumatoid arthritis: results of a randomized controlled trial. Arthritis Care Res (Hoboken).

