# Supplementary figures and images for: An Exercise and Educational and Self-management Program Delivered With a Smartphone App (CareHand) in Adults With Rheumatoid Arthritis of the Hands: Randomized Controlled Trial
Source: JMIR Mhealth Uhealth. 2022 Apr 7;10(4):e35462. doi: 10.2196/35462 (PMC9030995; doi:10.2196/35462)

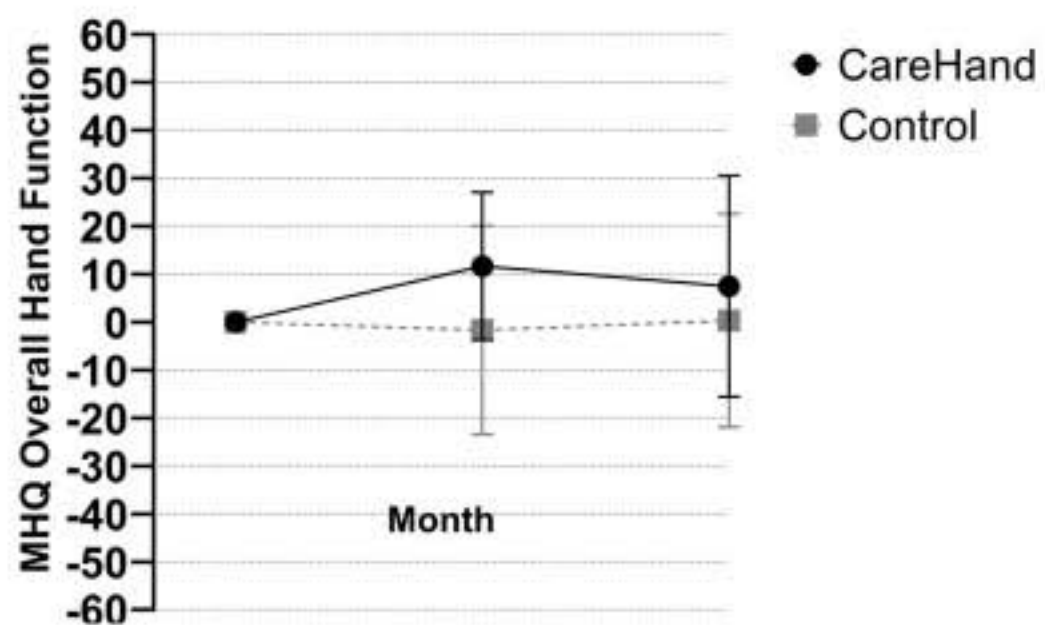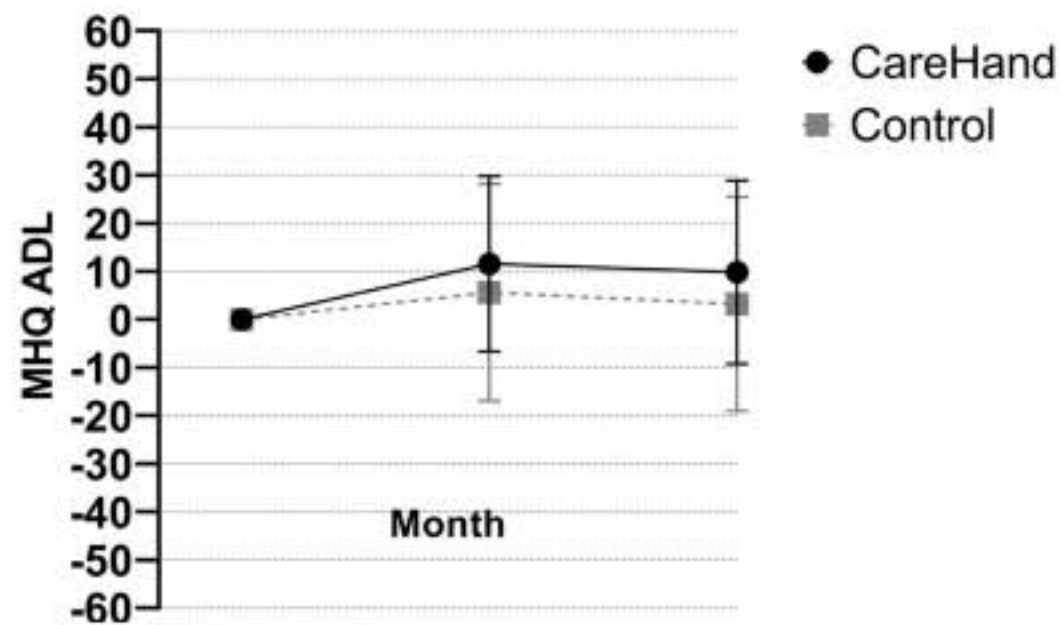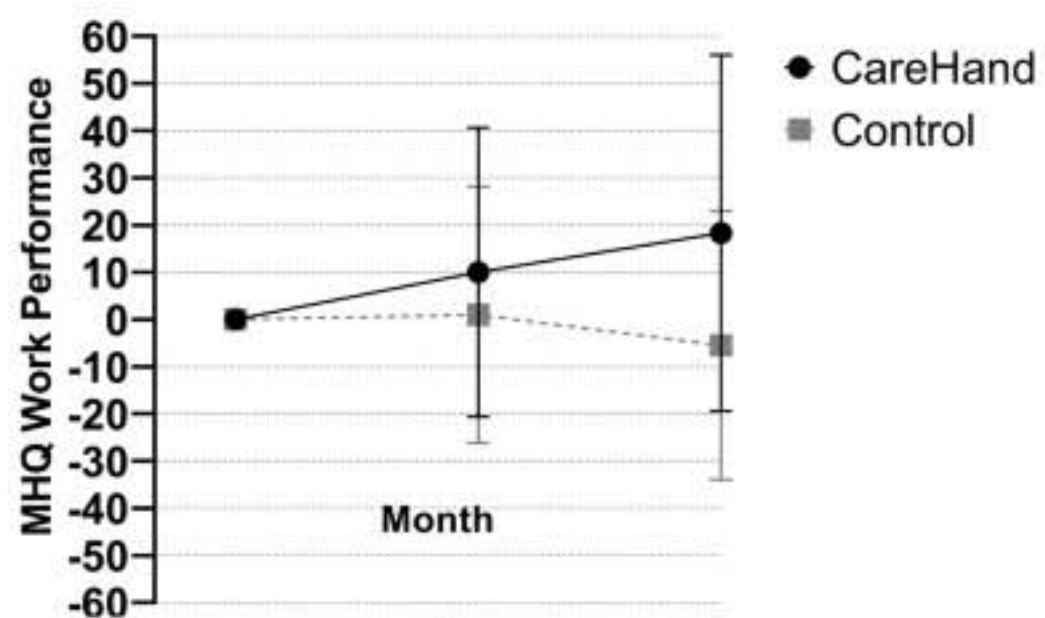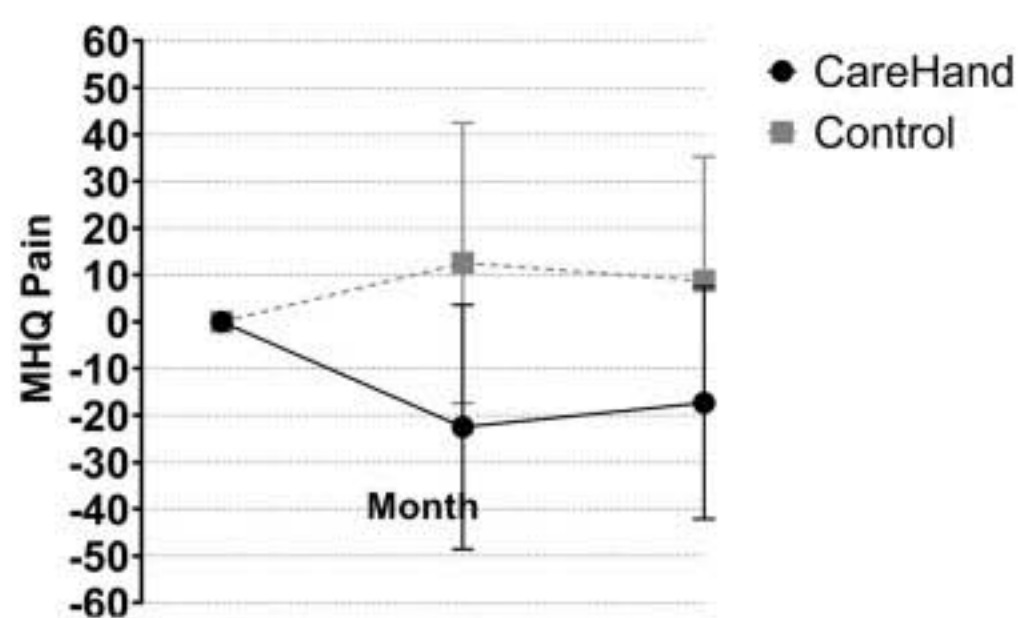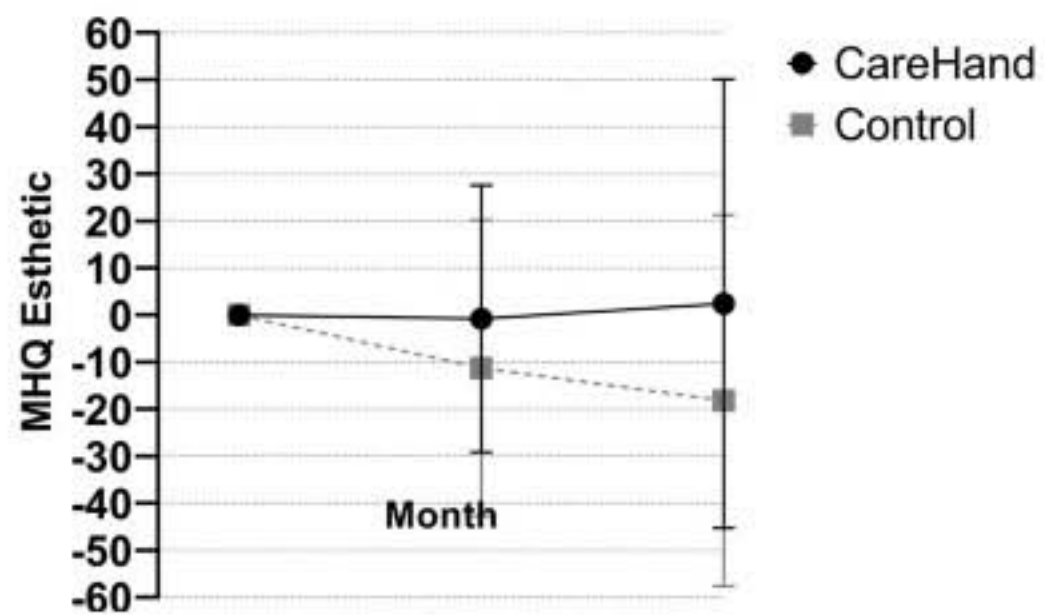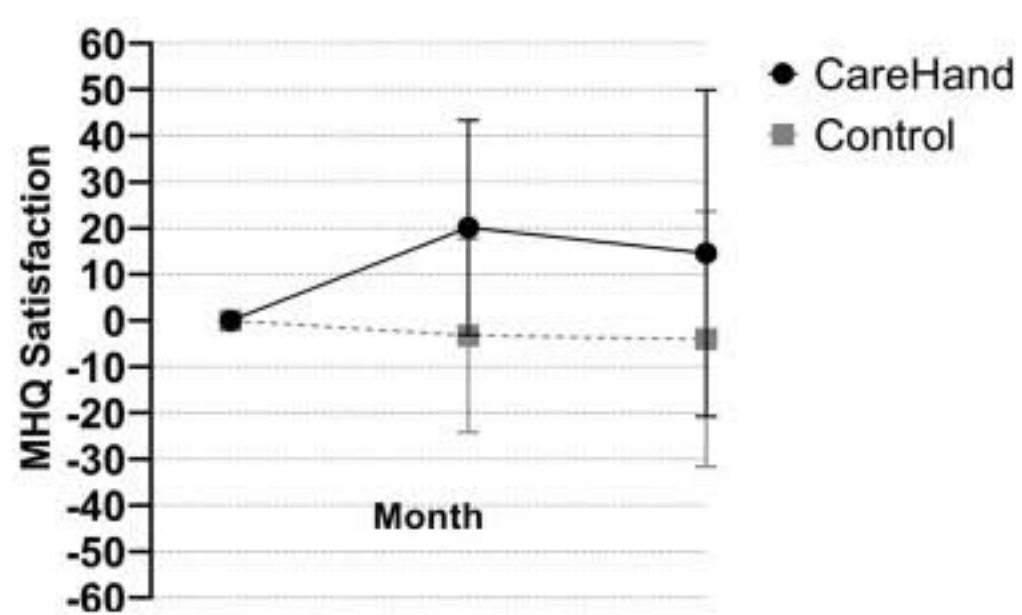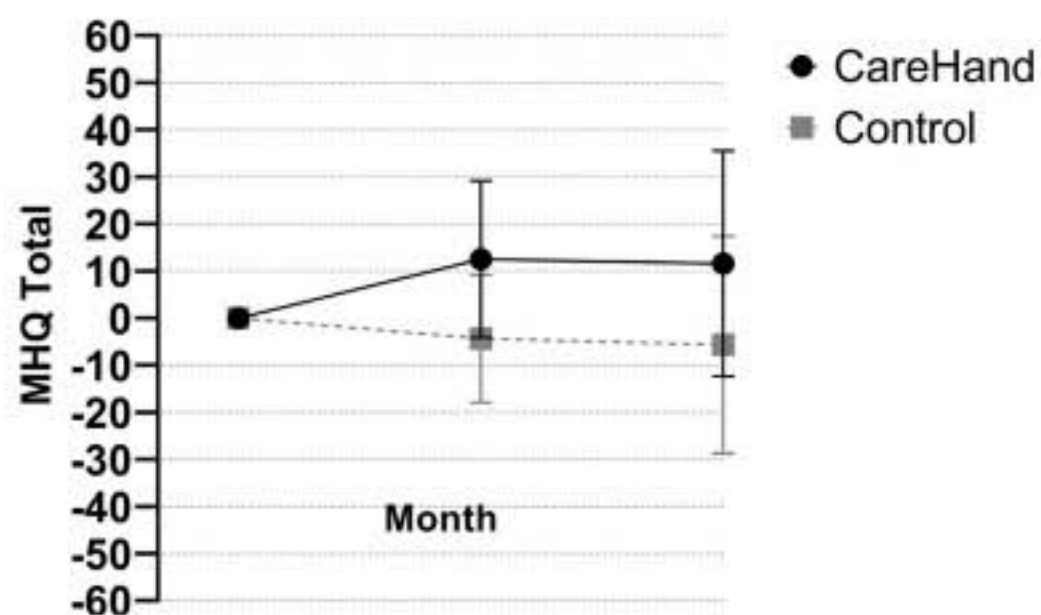

Supplement: Multimedia Appendix 1 [file mhealth_v10i4e35462_app1.pdf]
